# Anti-Inflammatory and Antioxidant Effects of ω-3 Polyunsaturated Fatty Acids on Astrocytes and Their Implications for the Blood–Brain Barrier’s Integrity and Function

**DOI:** 10.3390/ijms27062835

**Published:** 2026-03-20

**Authors:** Rimma Parnova, Ekaterina Fock

**Affiliations:** Sechenov Institute of Evolutionary Physiology and Biochemistry, Russian Academy of Sciences, 194223 St. Petersburg, Russia; efock@mail.ru

**Keywords:** ω-3 polyunsaturated fatty acids, docosahexaenoic acid, astrocytes, blood–brain barrier, brain endothelial cells, neuroinflammation

## Abstract

Impaired blood–brain barrier (BBB) integrity is a common hallmark of neurological disorders associated with neuroinflammation, neurodegeneration and aging. The function of the BBB relies heavily on the interaction between astrocytes and endothelial cells, the most closely connected elements of the neurovascular unit. Under inflammatory conditions, astrocytes can undergo a range of metabolic changes, becoming pro-inflammatory and harmful to endothelial cells. Upon activation, astrocytes secrete a plethora of inflammatory mediators that severely disrupt the barrier function of the BBB. ω-3 polyunsaturated fatty acids (PUFAs), mainly docosahexaenoic and eicosapentaenoic acids, exhibit protective anti-inflammatory and antioxidant effects demonstrated in various neurological disorders. This review focused on the role of ω-3 PUFAs and their oxidative derivatives, specialized pro-resolving mediators, in preserving the BBB’s integrity via suppression of astrocytes’ activation or even promotion of their transition from an A1 to an A2 phenotype. We considered mainstream mechanisms of the anti-inflammatory and antioxidant effects of ω-3 PUFAs on reactive astrocytes, such as stimulation of the Nrf2/ARE and Wnt/β-catenin signaling pathways, inhibition of NF-κB/matrix metalloproteinase activity and the JAK/STAT3 signaling axis, as well as the contribution of ω-3 PUFA-activated GPCRs and PPAR transcriptional factors, particularly regarding the role of these mechanisms in preserving the BBB’s integrity.

## 1. Introduction

Polyunsaturated fatty acids (PUFAs) from the ω-3 family, highly concentrated in the brain as components of membrane phospholipids, are essential at every step of brain development and contribute to numerous processes in the CNS. Docosahexaenoic acid 22:6ω3 (DHA), the end product of ω-3 PUFAs synthesis, is quantitatively the most important, while the levels of others, such as eicosapentaenoic acid 20:5ω3 (EPA), docosapentaenoic acid 22:5ω3 (DPA) and α-linolenic acid 18:3ω3 (ALA), are very low. A growing body of evidence indicates both independent and shared effects of these fatty acids in the CNS. DHA is essential for neuro- and synaptogenesis, neuronal differentiation, synaptic transmission and plasticity, neuronal network formation, energy metabolism, cerebral angiogenesis, clearance system, memory formation and behavior [1,2,3]. At the molecular level, DHA, along with other fatty acids, regulates gene expression and maintains optimal biophysical properties of the lipid bilayer, such as fluidity and flexibility, and lipid raft organization, which affects signal transduction pathways and the function of membrane proteins, such as receptors, transporters, enzymes and ion channels [4,5,6].

ω-3 PUFAs exhibit protective anti-inflammatory and antioxidant effects demonstrated in various neurological disorders, including ischemic injury [7,8], traumatic brain injury [9], Alzheimer’s disease [10], Parkinson’s disease [11], multiple sclerosis [12], epilepsy [13], depression [14], cognitive impairment [15] and aging. Moreover, EPA, DPA, and DHA can be metabolized into resolvins, protectins, and maresins, so-called specialized pro-resolving lipid mediators (SPMs), which actively resolve inflammation and promote tissue repair [16,17].

ω-3 PUFAs are essential for the development and function of the blood–brain barrier (BBB). Enrichment of the brain endothelial cell membrane in DHA has been shown to be necessary for the maintenance of low-caveolin-dependent transcytosis [18], for cerebral glucose uptake [19] and for clearance of amyloid-β from the brain [20]. In vivo and in vitro data demonstrate that DHA and EPA preserve and restore the properties of the BBB impaired in various pathological states [21,22,23,24,25,26]. Disruption of the BBB’s integrity, which is a common hallmark of neuroinflammation, neurodegeneration and aging, can be caused by numerous mechanisms. The crucial role belongs to the activation of astrocytes, the most abundant glial cell in the CNS, which, together with microvascular endothelial cells, pericytes, neurons, vascular smooth muscle cells and the vascular basement membrane, form the neurovascular unit [27,28].

It is plausible that the observed improvement of the BBB’s integrity under ω-3 PUFA supplementation can result from their anti-inflammatory and antioxidant effects on reactive astrocytes. The coordinated regulation of the BBB’s permeability is achieved through the close structural, functional and metabolic interactions that exist between astrocytes and endothelial cells of the microvasculature. Numerous astrocyte-derived protective paracrine mediators, such as Sonic hedgehog (Shh), transforming growth factor beta (TGF-β), glial-derived neurotrophic factor (GDNF), angiopoietin-1 and others, produced by astrocytes, control the maturation and stability of endothelial barrier function [29,30,31,32], as well as cerebral blood flow and angiogenesis. Nevertheless, under inflammatory conditions, astrocytes can undergo a range of functional and morphological changes, becoming pro-inflammatory and harmful to neighboring cells, including endothelial cells, which leads to the disruption of the integrity of the BBB [29,33,34,35,36].

The modulation of astrocyte activation along with improved neurological outcomes under dietary ω-3 PUFA supplementation was demonstrated in numerous pathological states, such as cerebral ischemia [37,38], Alzheimer’s disease [20,39], depression [40], sepsis [41], intoxication by neurotoxins [42], aging [43,44], and postoperative delirium-like behavior [22]. In accordance with these data, ω-3 PUFA deficiency is associated with increased astrogliosis [45].

This review focused on the effects of ω-3 PUFAs and their oxidative derivatives on the maintenance of the BBB’s integrity within astrocyte–endothelial cell interactions. We briefly described the mechanisms of ω-3 PUFAs transport across the BBB and their critical importance for DHA accumulation in the brain. Next, the signaling pathways known to be involved in the BBB’s disintegration associated with the pro-inflammatory state of astrocytes were considered. We presented evidence from in vitro and in vivo studies demonstrating the effects of ω-3 PUFAs or their derivatives on the suppression of astrocyte activation, highlighting the stimulation of Nrf2/ARE and the inhibition of NF-κB/matrix metalloproteinase (MMP) signaling pathways, the involvement of Wnt/β-catenin and JAK/STAT3 signaling cascades, PUFAs’ G-protein-coupled receptors, and the activation of PPAR transcriptional factors. Given the established close link between amyloid-beta (Aβ) pathogenesis, astrocyte activation, and BBB breakdown, we discussed the role of ω-3 PUFAs in the clearance of Aβ provided by the cooperative action of astrocytes and brain endothelial cells.

## 2. Reactive Astrocytes and Their Effects on BBB Integrity

The limitation of the entry of substances and immune cells into the brain tissue through the BBB depends mostly on two main structural peculiarities of the brain microvasculature: the presence of intercellular tight junctions (TJs) and adherens junctions (AJs) between adjacent endothelial cells, and the relatively low transepithelial permeability [28,46,47]. In addition, astrocytes form the so-called glial limitans, a barrier that restricts the penetration of large molecules and immune cells into the CNS parenchyma [48]. The BBB TJs consist of complexes formed by occludins, claudins (e.g., claudin-5), and junctional adhesion molecules (JAMS, e.g., JAM-A) that are linked to the endothelial cytoskeleton via zonula occludens proteins (e.g., ZO-1). Through their polarized endfeet, astrocytes enwrap the cerebral vascular wall. A wide array of receptors, transporters, and ion channels expressed in astrocytes allows them to control most properties of the BBB [31,49,50]. Coculturing of endothelial cells and astrocytes as an in vitro BBB model showed that astrocytes upregulate TJ protein levels [51,52], whereas genetic astrocyte ablation in the mouse brain significantly reduces TJ protein expression in the endothelium, which leads to an increase in BBB leakage [53]. Interestingly, astrocytes, by contacting their end processes, can induce barrier properties in non-neural endothelial cells [54].

Nevertheless, under pro-inflammatory conditions, the mechanisms by which astrocytes normally maintain the BBB’s integrity can be severely impaired, leading to the BBB’s disruption. CNS injuries are primarily responded to by microglia, the resident macrophages of the brain. Upon activation, microglia release IL-1α, TNF-α and complement component subunit 1q (C1q), which promote the generation of activated astrocytes. These astrocytes exhibit hypertrophy of their cell bodies and processes, accompanied by accumulation of glial fibrillary acidic protein (GFAP)-enriched intermediate filaments [55,56,57,58]. These morphological alterations are described as astrogliosis. Astrocytes that have undergone astrogliosis lose their regulatory and neuroprotective properties, including tripartite synapse functioning, thus contributing to synaptic dysfunctions, disruption of homeostasis and associated neuronal damage [59,60]. Many signal transducers, such as STAT3, NF-kB, JAK2, MAPK, PKC, Nrf2, mTOR, c-FOS and others, are implicated in the formation of reactive astrocytes [61]. The pro-inflammatory A1 and neuroprotective A2 phenotypes of reactive astrocytes have been identified in parallel with the M1 and M2 macrophages and microglia activation patterns [57]. However, transcriptomics has revealed a spectrum of astrocytic states that extends beyond the binary model, shaped by the influence of regional microenvironments and the type of injury [62]. Although pro-inflammatory microglia play a critical role in inducing A1-reactive astrocytes, in vitro and in vivo experiments evidence that astrocytes can also be activated independently on microglia [63,64,65].

Induction of the A1 astrocyte phenotype is considered to be a trigger for BBB impairment [34,35]. Reactive astrocytes produce a plethora of inflammatory mediators, such as IL-1β, TNF-α, and IL-6 [33,66], ROS, IFN-γ, and TGF-β [67], and complement system components [68], which severely disrupt the barrier function of the BBB through various mechanisms, including alterations in the localization and expression of TJ proteins, endothelial cells apoptosis, degradation of the glycocalyx, the increase in transcytosis and others [33,69,70]. For example, sleep restriction promotes a chronic low-grade inflammatory status in the cortex and hippocampus. It is associated with high levels of TNF-α, IL-1β, and IL-6, a progressive increase in BBB permeability and TJ disassembly, and a higher expression of GFAP and C3 complement component (A1 astrocytes marker) [71]. Reactive astrocytes, as well as microglia and endothelial cells, secrete numerous mediators, including MMP9 and MMP2, which induce the rearrangement and degradation of TJ proteins and facilitate BBB dysfunction, allowing leukocyte infiltration and amplifying inflammation [72,73,74,75,76,77]. MMP2 promotes BBB disruption by blocking the interaction between astrocytes and endothelial cells via inhibiting the Shh pathway, as was shown in early cerebral ischemia [78]. Therefore, the mechanisms by which astrocytes support the integrity of the BBB can be severely compromised during acute and chronic inflammation. MMP9 is also responsible for brain endothelial cells’ apoptosis by disturbing cell–matrix interactions [79]. Activated A1 astrocytes produce cell adhesion molecules (ICAM-1 and VCAM-1) and release CXCL1, CCL20 and CCL2 chemokines, promoting the increased penetration of peripheral immune cells across the BBB, thereby exacerbating BBB disruption and neuroinflammation [80,81,82,83]. Vascular endothelial growth factor (VEGF) is among the identified paracrine substances secreted by astrocytes, which contribute to the BBB’s breakdown through multiple mechanisms [31,84].

In contrast, a neuroprotective A2 astrocyte phenotype preserves the BBB’s integrity by suppressing the pro-inflammatory responses via inhibition of NF-κB signaling pathways [23,85] and promotion of the expression of anti-inflammatory cytokines, such as TGF-β, IL-4, IL-10, and IL-13, through the activation of the Nrf2 pathway [67,86]. A2 reactive astrocytes produce various neurotrophic factors [30,31,87], such as glial-derived neurotrophic factor (GDNF) [88], which increases claudin-5, occludin, and ZO-1 expression [89]. Selective ablation of the A2 astrocyte phenotype compromises BBB integrity, increases the infiltration of peripheral immune cells, and causes an inability to restore the BBB [90].

Meanwhile, the endothelial cell response, which may be a consequence of systemic inflammation associated with a significant increase in inflammatory cytokine levels in the bloodstream [70], can activate glial cells and become a driver of neuroinflammation. The infiltrating leukocytes secrete MMPs, which target components of the basement membrane and astrocyte endfeet, leading to the activation of the reactive gliotic program [29]. Brain endothelial cells have been found to release microvesicles that affect astrocytes. Microvesicles obtained from endothelial cells cultured in oxygen/glucose deprivation promoted astrocyte apoptosis by upregulating caspase-9 and downregulating the anti-apoptotic protein Bcl-2 [91].

Under inflammatory stimuli, brain endothelial cells upregulate the expression of A1 astrocytic genes [92]. Interestingly, the transcriptome of astrocytes activated by endothelial cells or microglia differs, indicating multiple phenotypes of activated astrocytes [92].

## 3. Transport of PUFAs Across BBB

The most common model in ω-3 research is a dietary supplement consisting of a mixture of unesterified EPA and DHA, or fish oil containing both fatty acids. These dietary interventions typically last for a long time (several weeks or longer). Although the bioavailability of these dietary forms of ω-3 PUFAs for the brain is debated [93], there is considerable evidence that dietary supplementation with EPA + DHA, either as free fatty acids or as components of fish oil, increases the content of DHA, DPA and EPA, and decreases the ω-6/ω-3 ratio in brain tissue [26,39,42,43,94,95]. Culturing brain cells with DHA and EPA results in a multiple increase in their concentration in plasma membranes, while DHA is more than twice as likely as EPA to be incorporated into membrane lipids [96,97].

**Figure 1:**
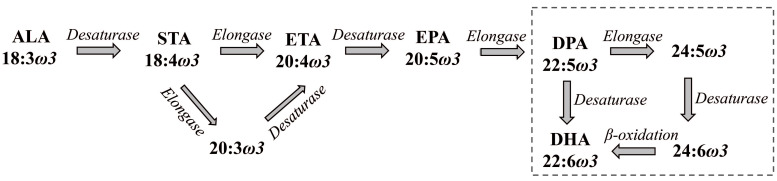
A scheme of biosynthetic pathways of ω-3 PUFAs. STA—stearidonic acid; ETA—eicosatetraenoic acid. For others, refer to the list of abbreviations. The dotted line represents the microsomal–peroxisomal coupled pathway, also known as Sprecher’s shunt.

The main source of DHA in the brain is its pool in the bloodstream, either in albumin-complexed form or esterified in plasma lipids. Although astrocytes can convert ALA to DHA [98,99,100], which is important for supplying neurons, the contribution of this biosynthesis to the overall DHA pool in the brain appears to be minor. The biosynthetic pathways of ω-3 PUFAs are presented in Figure 1.

The delivery of either dietary or liver-produced ω-3 PUFAs to the brain is critically dependent on the PUFA-transporting abilities of the endothelial cells of the BBB. The transport of PUFAs through the BBB may occur either by passive diffusion or by fatty acid transporter proteins (FATPs), which facilitate PUFAs’ transfer across the membrane. Inside endothelial cells, the trafficking of DHA is governed by fatty acid-binding proteins (FABPs), which interact with the hydrophobic long chain of PUFAs. Brain microvascular endothelial cells express FATP1, FATP4, fatty acid translocase/CD36, and fatty acid-binding proteins (FABPs), such as FABP3, FABP4 and FABP5, which facilitate PUFA trafficking intracellularly [101,102,103]. LysoPC-conjugated DHA has been shown to be a predominant form of its delivery to the brain [93,104]. LysoPC is specifically transferred by the sodium-dependent transporter Mfsd2a, which is highly expressed in the endothelium of the BBB and is considered a key player in the delivery of DHA to the brain [105].

Impaired transport of DHA across the BBB is one of the identified reasons for the decreased levels of DHA in brain cells. For instance, in the mouse model of Alzheimer’s disease (APP/PS1 mice), reduced brain levels of DHA have been shown to be associated with decreased expression of FABP5 in brain microvessels [106], or FATP1, as was shown in vitro in cerebral microvascular endothelial cells treated with Aβ_25-35_ [107]. Silencing of FABP5, both in vitro and in vivo, led to significant reductions in transport of ^14^C-DHA through the BBB and, subsequently, endogenous brain DHA levels [108]. A 50% reduction in DHA content was observed in the brains of mice lacking Mfsd2a and, therefore, unable to take up DHA-containing LysoPC across the BBB [105]. Notably, DHA enhances its own uptake by the brain by upregulating the expression of fatty acid-binding and -transporting proteins, as shown for FABP5, FATP1, and FATP4 in the brain endothelium [103] and for Mfsd2a in the retinal vasculature [109].

There is evidence suggesting a link between DHA deficiency in the brain and astrocyte activation. For instance, aging-induced neuroinflammation and increased astrogliosis correlate with decreased endogenous DHA levels in the brain, suggesting impaired DHA transport across the BBB [45]. DHA deficiency may lead to the enhancement of arachidonic acid (ARA) levels and a subsequent increase in the ARA-induced pro-inflammatory cascade, leading, in turn, to astrogliosis. Decreased brain DHA levels and increased astrogliosis were also demonstrated in mice deficient in acyl-CoA synthetase 6, a member of the acyl-CoA synthetase family, which has been shown to be necessary for brain DHA enrichment [110].

## 4. Anti-Inflammatory and Antioxidant Effects of ω-3 PUFAs on Astrocytes

Dietary supplementation of ω-3 PUFAs has been shown to reduce the activation of astrocytes and microglia, decrease the production of pro-inflammatory cytokines and improve neurological outcomes, as was demonstrated in multiple pathological models, such as cerebral ischemia [37,38,111], Alzheimer’s disease [20,39], Parkinson’s disease [112], depression sepsis [41], neurotoxin-induced intoxication [42], aging [43,44,45], and intracerebroventricular injection of IL-1β [94]. When EPA and DHA are added separately, their effects on activation of astrocytes might be different from each other depending on the specificity of neuropathology. For instance, EPA is well known to be superior to DHA in the treatment of depression [113]. Consistent with this, in a mouse model of depressive-like behaviors, the effect of EPA on inhibition of activated astrocytes and the associated signaling pathways was more pronounced than that of DHA [40]. In contrast with that, DHA was more effective than EPA in alleviating aging-associated neuroimmunological changes [44].

Astrocyte activation is usually evaluated by an increase in GFAP or, in some cases, calcium-binding protein B (S100B) and complement component 3 (C3) expression levels, which are the markers of the A1 pro-inflammatory astrocyte phenotype associated with astrogliosis. Elevated levels of pro-inflammatory cytokines in brain tissue, such as TNF-α, IL-1β, and IL-6, are largely regarded as indicators of astrocytes and microglia activation, signifying a prevailing pro-inflammatory phenotype. To a lesser extent, brain endothelial cells also contribute to the production of pro-inflammatory cytokines in the brain.

The role of ω-3 PUFAs in the promotion of a transition of the astrocyte phenotype from A1 to A2 was established in the rat model of aging [44]. In the hippocampus of old rats in comparison with young animals, the mRNAs of *GFAP*, *S100B* and *C3* (A1 markers) were significantly upregulated, while the mRNA of A2 marker *S100A10* was markedly downregulated. Dietary supplementation with EPA and DHA equally inhibited the increase in A1 markers and rise in TNF-α, IL-1β, and IL-6 levels in the hippocampus, which signified a prevailing A1-like pro-inflammatory phenotype. The decrease in mRNA expression of *S100A10* was markedly reversed by DHA treatment, but not EPA [44]. Similar differences were found in the effect of DHA and EPA on M1- and M2-specific microglia markers. The authors suggested that the imbalance between the two types of glial polarizations is associated with aging-induced memory impairment. BBB permeability was not studied in that work, but we suggest that the suppression of pro-inflammatory cytokine production under ω-3 PUFA supplementation should restore barrier properties.

In another study, a simultaneous upregulation of the expression of both A1 and A2 markers (C3 and S100a10, respectively) was found in the hippocampuses of mice treated with the neurotoxin trimethyltin, a drug widely used in industry and agriculture. Flaxseed oil, which lacks C20-22 PUFAs but is enriched in ALA, a precursor in EPA and DHA synthesis, prevented a neurotoxin-induced increase in *GFAP* and *C3* mRNAs in the hippocampus. Surprisingly, the level of *S100a10* mRNA, an A2 marker, was also increased under the neurotoxin treatment and was inhibited by flaxseed oil. In contrast with in vivo data, in primary cultured astrocytes, ALA prevented the effect of neurotoxin on downregulation of A2 marker (S100a10) and upregulation of A1 marker (C3) and efficiently suppressed the production of inflammatory mediators. These data indicate that ALA maintains the protective astrocyte phenotype [42]. However, it remains unclear whether ALA acts on its own or via its elongation/desaturation products, since ALA can be converted into EPA/DHA in astrocytes [99].

In the mouse model of transient middle cerebral artery occlusion, ω-3 PUFAs reduced stroke-induced A1 astrocyte polarization and improved functional outcomes [37]. The same result was found in vitro in astrocyte primary cultures subjected to hypoxic conditions. DHA treatment limited A1-specific astrocyte polarization manifested by the reduction in elevated levels of A1 markers (*Amigo2*, *H2-D1*, *H2-T23*, *Serping1*, and *Ugt1a*). However, in these experiments, DHA did not change the levels of A2-specific markers (*B3gmt5*, *CD14*, *Emp1*, and *Slc10a6*) [37].

The restoration of low BBB permeability and TJ protein expression, along with altering astrocytic activity under ω-3 PUFA dietary supplementation, was demonstrated in animal models of uremic brain injury [21], hypoxic–ischemic brain injury [114], Alzheimer’s disease [26], traumatic brain injury [25], and postoperative delirium-like behavior [22].

ω-3 PUFAs’ effect on BBB properties impaired by IL-1β was studied in vitro on a “BBB-on-chip model” consisting of human brain microvascular endothelial cells (hBMECs), human astrocytes, and human brain vascular pericytes. It was found that pretreatment with ω-3 PUFAs rescued disruption of the astrocyte network density caused by IL-1β, protected barrier permeability, manifested as restoration of the TEER value and ZO-1 expression, and decreased VCAM-1 overexpressed in both endothelial cells and pericytes [22]. Coculturing of primary mouse brain endothelial cells and astrocytes revealed that ω-3 PUFAs (DHA + EPA) promote endothelial cell proliferation and increase the expression of adherens junctional proteins, thereby improving the barrier formation. Importantly, the DHA/EPA treatment did not affect endothelial cell barrier formation in the absence of astrocytes, indicating that astrocytes are the primary sensor of ω-3 PUFAs, at least in vitro [115].

One of the most important consequences of astrocyte activation on the integrity of the BBB is the increased production of pro-inflammatory cytokines, chemokines, ROS, NO, and prostaglandins, which trigger inflammation and promote BBB impairment. Numerous studies on in vivo models and on astrocyte primary cultures or cell lines have shown that ω-3 PUFAs improve antioxidant status and downregulate the release of inflammatory mediators [38,41,42,43,44,63]. This effect is based on the activation of the antioxidant defense mediated by the redox-sensitive nuclear factor erythroid 2-related factor 2 (Nrf2) and on inhibition of the pro-inflammatory pathway mediated by NF-kB [86,116,117]. In astrocytes, as in many other cell types, both pathways are closely connected to each other by a complex signaling network [118,119,120]. Thus, activation of NF-κB and expression of pro-inflammatory cytokines, as well as augmentation of MMP9, were significantly more pronounced in primary cultured astrocytes isolated from Nrf2-knockout mice than in wild-type mice [118,119].

**Figure 2:**
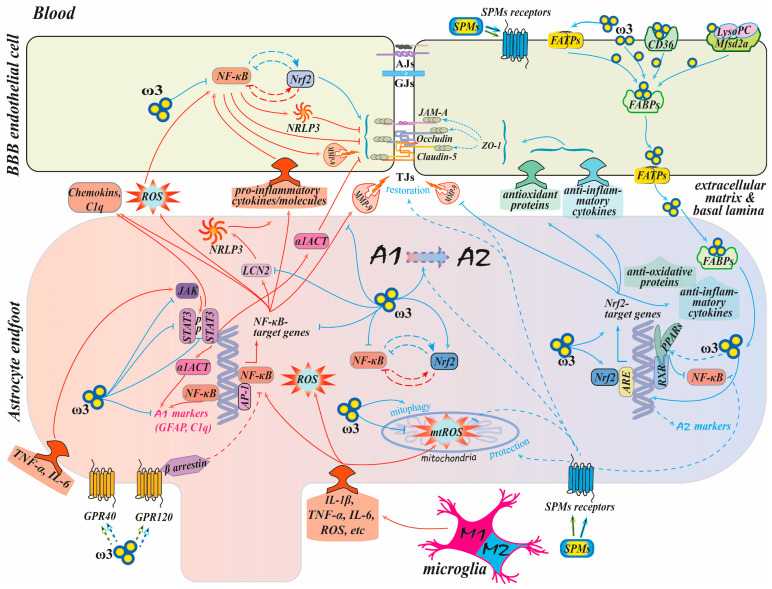
The anti-inflammatory and antioxidant effects of ω-3 PUFAs on astrocytes and their impact on brain endothelial cells. For details, see the text. Briefly, pro-inflammatory stimuli increase the production of pro-inflammatory mediators by activating the NF-κB nuclear factor, which results in the expression of NF-κB target genes. This leads to the disruption of TJ proteins and compromises the barrier integrity. ω-3 PUFAs exert their anti-inflammatory and antioxidant effects in astrocytes both through receptors and directly, attacking their targets inside the cell. ω-3 PUFAs inhibit the shift of astrocytes towards a pro-inflammatory phenotype by activating the Nrf2/ARE signaling pathways. This activation suppresses NF-κB activity, reduces the expression of pro-inflammatory agents, and enhances the production of antioxidative proteins and anti-inflammatory cytokines, promoting the restoration of TJ proteins and BBB integrity. ω-3 PUFAs can counteract the JAK2/STAT3 signaling pathway, thereby inhibiting the inflammatory response of astrocytes. The solid lines represent established pathways, whereas the dotted lines depict potential mechanisms. Red arrows signify a damaging scenario, and blue arrows indicate a pro-resolving one. For symbols in the scheme, see the list of abbreviations.

In the following sections of this review, we will discuss in detail the available data on the mechanisms mediating the effect of ω-3 PUFAs on astrocytes, particularly regarding their role in preserving the BBB’s integrity. The described signaling pathways are illustrated schematically in Figure 2.

### 4.1. Nrf2/ARE Pathway

Nrf2 is a key transcription factor that plays a pivotal role in cellular antioxidant and detoxifying responses. Under basal conditions, Nrf2 is associated with its cytosolic inhibitor, Kelch-like ECH-associated protein 1 (Keap1). Following oxidative stress or other damage insults, the Nrf2 protein dissociates from the Keap1 homodimer, translocates to the nucleus, and binds to the antioxidant response element (ARE) sequences within the promoter regions of target genes. These genes are involved in antioxidant defense, glutathione (GSH) production and regeneration, NADPH restoration, and many other processes [86,117]. Nrf2 is highly expressed in astrocytes [63,97,118].

In rat primary astrocytes subjected to H_2_O_2_-induced oxidative stress, EPA and DHA at micromolar concentrations decreased cytotoxicity, reduced ROS levels and increased expression of Nrf2, glutamate cysteine ligase (GCL), glutathione synthetase (GS), and glutathione peroxidase 4 (GPx4) [97,121]. Rat primary glial cell culture, enriched in astrocytes, derived from the rats that received DHA intraperitoneally prior to stroke injury, demonstrated upregulation of heme oxygenase 1 (HO-1) expression, another key Nrf2 target gene involved in cytoprotection against oxidative stress [111]. EPA pretreatment of the Gibco^®^ Human Astrocytes cell line effectively protected the cells from H_2_O_2_ insult. In these experiments, EPA withstood the cytotoxicity and oxidative stress, restored GSH levels and enhanced the expression of Nrf2, HO-1, and NADPH:quinone oxidoreductase 1 (NQO-1) proteins, which mitigate the oxidative stress response [121].

Although Nrf2 is expressed in a variety of CNS cells, some data indicate a specific role of astrocyte-localized Nrf2 in the improvement of neurological outcomes. Thus, ketamine, a rapid-acting antidepressant, has been found to alleviate anxiety- and depression-like behaviors in mice by upregulating Nrf2 specifically in astrocytes. This upregulation promotes the expression of antioxidant enzymes, such as HO-1, NQO-1, and SOD, suppresses the production of astrocyte-derived pro-inflammatory cytokines, including iNOS, TNF-α, and IL-6, and enhances the expression of anti-inflammatory factors like IL-10 and TGF-β [65].

In primary cerebral cortex astrocytes subjected to oxygen-glucose deprivation, DHA reduced A1 astrocyte polarization, attenuated mitochondrial oxidative stress and mitochondrial ROS production, and promoted mitophagy and mitochondrial fusion under hypoxic conditions [37]. The proposed mechanisms of DHA’s effect seem to be related to mitochondrial phospholipid remodeling by increasing the content of cardiolipin, a tetra-acyl phospholipid, in the mitochondrial inner membrane [37,122].

### 4.2. NF-kB and MMPs

In parallel with Nrf2 activation, the inhibition of NF-kB and the downstream inflammatory cascade is a current strategy to counter the neuroinflammation afforded by ω-3 PUFAs. NF-κB cascade is induced in response to oxidative stress and participates in complex inflammatory pathways, activating the production and release of pro-inflammatory cytokines in many types of cells, including astrocytes. An increasing amount of evidence indicates that ω-3 PUFAs inhibit NF-kB activation in astrocytes [42,116]. For example, in primary cultured astrocytes treated with IL-1β, DHA inhibited the translocation of the p65 subunit of NF-κB to the nucleus, decreased the p65 protein level in the nucleus, and reduced dose-dependently IκB protein phosphorylation and the binding of the AP-1 transcription factor members (c-Jun/c-Fos) to DNA. This led to the downregulation of pro-inflammatory TNF-α and IL-6 cytokines’ secretion and the dampening of the expression of pro-inflammatory proteins, such as COX-2 and iNOS [116]. ω-3 PUFA-induced inhibition of the expression of COX-2, iNOS, and hypoxia-inducible factor 1alpha and IL-1β was also observed in primary astroglia cultures exposed to hypoxia [38]. These data are in accordance with the observed reduction in COX-2-produced pro-inflammatory prostaglandins (i.e., PGE_2_ and PGF_2_α) levels in astrocytes due to the increased release of endogenous DHA from phospholipids driven by VIB Ca^2+^-independent phospholipase A2 [123].

It is well established that reactive astrocytes produce MMP2, MMP3 and MMP9 in an NF-κB-dependent manner [124]. The promoter regions of MMP genes typically contain binding sites for AP-1 and NF-κβ transcription factors [125,126]. Elevated MMP9 levels are strongly associated with BBB disruption through the degradation of TJ proteins, the extracellular matrix, and the basement membrane [23,72,73,127]. ω-3 PUFAs suppress MMP9 expression and activity, which has been widely demonstrated in numerous in vivo and in vitro inflammatory models, as well as in clinical studies [23]. It is suggested that ω-3 PUFAs can reduce MMP9 synthesis by suppressing AP-1 and NF-κB, which are attached to the *MMP9* gene promoter [23,125].

In a rat model of traumatic brain injury, DHA has been found to inhibit edema formation and enhance endothelial occludin expression via a suppression of MMP9 expression in activated astrocytes surrounding the cerebral blood vessels [25]. The use of a selective antagonist of the G protein-coupled receptor GPR120, whose endogenous ligands are medium- and long-chain PUFAs, revealed that the inhibition of GPR120 signaling increased the levels of *Mmp2* and *Mmp14* mRNAs, and decreased the expression levels of tissue inhibitor of metalloproteinases 3 (*Timp3*) and *Timp4*, suggesting that GPR120 negatively regulates the astrocyte-derived MMP network [128].

DHA and EPA regulate the functioning of the immunoproteasome (iP), a pivotal player in innate and adaptive immune responses to pro-inflammatory and oxidative stress insults. For instance, in astrocytes, Aβ has been shown to enhance the proteasome activity and increase the expression of iP subunits [129]. It was demonstrated that DHA and EPA suppress the expression of IL-1β-induced iP subunits in primary astrocytes [130]. The promoter of the iP subunit genes has binding sites for the NF-κB dimer [130]. Since DHA has been shown to dampen NF-κB activation in astrocytes [116], it is reasonable to speculate that DHA-induced inhibition of iP subunit expression is mediated, at least in part, by DHA-induced suppression of NF-κB.

### 4.3. JAK/STAT3 Pathway

The JAK/STAT3 signaling pathway is associated with the induction of astrogliosis and the progression of neurodegenerative diseases in response to injury [42,56,131,132]. The selective deletion of STAT3 from astrocytes in STAT3 conditional knockout mice markedly attenuated neurotoxicity-induced astrogliosis [132]. Activation of the JAK2/STAT3 pathway in reactive astrocytes contributes to the BBB’s disruption in pathological conditions [133,134]. STAT3-dependent expression of Serpina3n (encoding alpha 1-antichymotrypsin) was identified as a factor derived from astrocytes that causes BBB disruption [36].

The involvement of the JAK2/STAT3 signaling pathway in the protective effects of ω-3 PUFAs and SPMs has been demonstrated in a wide range of cells and tissues [135,136,137,138], indicating that this mechanism of ω-3 PUFAs’ action is likely conserved among different cell types. In primary cultured astrocytes, upregulation of *Jak2*- and *Stat3*-mRNA induced by the neurotoxin trimethyltin was fully prevented by ALA treatment [42]. Although the data are limited, it is highly likely that ω-3 PUFAs or SPMs counteract the JAK2/STAT3 signaling pathway in astrocytes, thereby inhibiting the inflammatory response.

### 4.4. LCN2-NLRP3 Signaling Axis

ω-3 PUFAs can affect astrocytes’ activation and the BBB’s integrity via suppression of the LCN2-NLRP3 signaling axis. The NOD-, LRR- and pyrin domain-containing protein 3 (NLRP3) inflammasome, a key component of the innate immune system, operates downstream of NF-κB and amplifies the inflammatory response, primarily by promoting the maturation of IL-1β, IL-18, and caspase-1 [139]. NLRP3 is present in astroglia [140,141,142]. In mouse primary astrocyte culture, NLRP3-caspase-1 inflammasome activation has been shown to mediate Aβ-induced IL-1β expression [141]. NLRP3 is also expressed in the brain microvascular endothelial cells and mediates the injury-induced increase in BBB permeability and downregulation of *ZO-1* gene expression [143,144].

Lipocalin 2 (LCN2), a protein with multiple biological functions, including innate immune and inflammatory response, is primarily released by, and also acts on, glial cells to promote neuroinflammation under brain injury conditions [145]. Its expression increases significantly after acute injuries and during chronic neuroinflammation [146,147]. The released LCN2 amplifies inflammation and activates both the microglia and astrocytes in a para- and autocrine manner, acting as a molecular switch, which determines the phenotypic type of the astrocytes, shifting it towards A1. Expressed both in astrocytes and endothelial cells, LCN2 promotes BBB disruption [146,147,148] and causes astrocyte pyroptosis through an autocrine mechanism by activating the NLRP3 inflammasome [149]. NF-kB plays a dual role in LCN2 signaling pathways. It upregulates the expression of the *LCN2* gene, and, meanwhile, is a part of the downstream signaling pathways activated by LCN2 [145].

The downregulation of *Lcn2*-mRNA and the suppression of astrocyte activation by supplementation of ALA were demonstrated in primary astrocytes exposed to the neurotoxin trimethyltin [42]. In a mouse model of depressive-like behaviors, the beneficial effect of EPA or DHA was shown to be associated with the inhibition of astrocyte activation, mediated by the suppression of the LCN2-NLRP3 signaling pathway [40]. However, it remains unclear whether this mechanism of EPA/DHA protection is common to other neuropathologies.

### 4.5. Wnt/β-Catenin Signaling Cascade

ω-3 PUFAs activate the canonical Wnt signaling cascade within astrocytes [150]. This signaling pathway specifically regulates brain angiogenesis and barriergenesis [151] and preserves the function of brain cells, including astrocytes and cerebral endothelial cells. Astrocyte-derived Wnts maintain Wnt/β-catenin activity either in endothelia or astrocytes, thereby controlling transcytosis and vesicular abundance in endothelial cells, as well as endfeet integrity in astrocytes [152]. Wnt/β-catenin activity is suppressed in a wide range of disease scenarios, leading to enhanced oxidative stress and increased cellular apoptosis that can result in a dysfunctional BBB [31]. The Wnt/β-catenin signaling mediates the effect of astrocyte-secreted Shh on the increase in TJ proteins and junctional adhesion proteins in endothelial cells [153].

In primary astrocytes, the activation of the Wnt/β-catenin signaling pathway has been shown to mediate the antioxidant effects of DHA/EPA against isoflurane-induced toxicity. Through partial reactivation of the canonical Wnt pathway, these fatty acids stimulated SOD activity, dampened ROS production and ameliorated gap junction–connexin Cx43 uncoupling, thereby restoring astrocytic gap junction function induced by injury [150].

### 4.6. GPR120

GPR120 (or FFAR4) is a G-protein-coupled receptor, which specifically binds medium- and long-chain free fatty acids. It is expressed in astrocytes [128,154,155], as well as in microglia [154,155,156], and in peripheral immune cells [157,158]. GPR120 has been shown to be markedly upregulated in astrocytes, but not in neurons or microglia, within the spinal cord following injury [155].

The involvement of GPR120-mediated signaling in astrocytic Aβ-degrading activity has been shown with the use of AH7614, a GPR120-selective antagonist. It turned out that GPR120 negatively regulates the astrocyte-derived MMP2 and MMP14, the degrading enzymes for extracellular Aβ. However, neither DHA nor CpdA, another GPR120-selective antagonist, had any effect [128].

GPR120-mediated signaling in inflammatory conditions defined in other cells shares a common pathway that includes β-arrestin 2/IkappaB kinase/NF-κB steps [154,158,159]. Although limited data are available, it is possible to suggest that ω-3PUFA-induced inhibition of NF-κB in astrocytes may be triggered by the activation of GPR120. GPR40, another fatty acid receptor, has also been detected in astrocytes, as well as in microglia and neurons [160]; however, its specific role in astrocytes has not been revealed yet.

### 4.7. PIEZO1 Cation Channel

One more mechanism of ω-3 PUFAs’ action on astrocytes activation could be related to their potential effect on Piezo1, a mechanosensitive, non-selective cation channel whose gating is primarily regulated by surrounding lipids and the lipid bilayer’s physical properties [161,162,163,164]. Astrocytes are highly mechanosensitive cells that detect changes in the mechanical properties of damaged, aging, or degenerating brain tissue [165,166]. Piezo1 is expressed in astrocytes [163,164] and is considered an important determinant of the pathogenesis of neuroinflammation via activation of Ca^2+^ influx and NF-κB p65 signaling transduction [165,166,167]. The role of Piezo1 was revealed in the LPS-induced mouse model of neuroinflammation [41]. It was found that EPA/DHA-induced suppression of astrocytes and microglia activation in the hippocampus and the inhibition of pro-inflammatory cytokine production depend on the downregulation of *Piezo1* mRNA and protein expression. This effect was mediated by miR-107, a small non-coding RNA, whose expression was upregulated by EPA/DHA supplementation [41].

### 4.8. PPARs/RXR Signaling

Free fatty acids and their derivatives are endogenous ligands for peroxisome proliferator-activated nuclear receptors (PPARs), which function as transcription factors and control the expression of numerous genes involved in the regulation of lipids of biosynthesis and glucose metabolism, as well as in cell development. When activated, the PPAR heterodimerizes with the retinoid X receptor (RXR) to interact with the promoter region of target genes for their expression. PPAR ligands, in particular, those of PPARα and PPARγ, inhibit the activation of pro-inflammatory gene expression and can negatively interfere with pro-inflammatory transcription factor signaling pathways in vascular and inflammatory cells [168]. The triad of PPARα, PPARβ/δ, and PPARγ, as well as RXRα, is expressed in astrocytes [169,170], and all of them are implicated in concert in regulation of astrocyte responses to inflammatory stimuli. PPARα activation by fibrates was shown to prevent astrogliosis and neuroinflammation by inhibiting NF-kB-DNA-binding activity [170,171,172]. In primary rat astrocytes exposed to LPS, synthetic ligands for PPARα, PPARβ or PPARγ decreased the levels of the pro-inflammatory cytokine TNFα and COX-derived oxylipins, whereas the PPARβ agonist stimulated the production of pro-resolution mediators, derivatives of DHA [169]. The PPARβ/δ agonists were able to protect astrocytes against oxidative stress by enhancing degradation of peroxisomes [173].

The PPARγ/NF-κB pathway has been shown to mediate DHA-induced inhibition of microglia activation [174]. To our knowledge, despite the high level of PPAR expression, no direct data on the contribution of PPARs to the effects of ω-3 PUFAs in astrocytes were found. However, Needham and coauthors [175] proposed coupling of DHA and arachidonic acid (ARA) to PPARγ in astrocytes, which can lead to activation of anti-inflammatory or pro-inflammatory effects depending on the ratio of ω-3/ω-6. The key player in this dichotomy is the protein FABP7 (fatty acid-binding protein 7), which not only binds fatty acids in the cytosol but also is endowed with its own signaling functions [176]. FABP7 is highly expressed in astrocytes in the developing and mature brain [175,177] and is critically important for astrocyte lipid homeostasis, neuronal excitatory synapse formation, and synaptic transmission [178]. It was hypothesized that higher relative levels of DHA promote FABP7-mediated transfer of DHA to the nucleus, resulting in the subsequent activation of PPARγ and the transcriptional activation of anti-inflammatory pathways. Alternatively, higher relative levels of ARA promote FABP7-mediated delivery of ARA to the endoplasmic reticulum to interact with COX-2, which generates pro-inflammatory prostaglandins [175].

## 5. ω-3 PUFA-Derived Pro-Resolving Lipid Mediators

**Figure 3:**
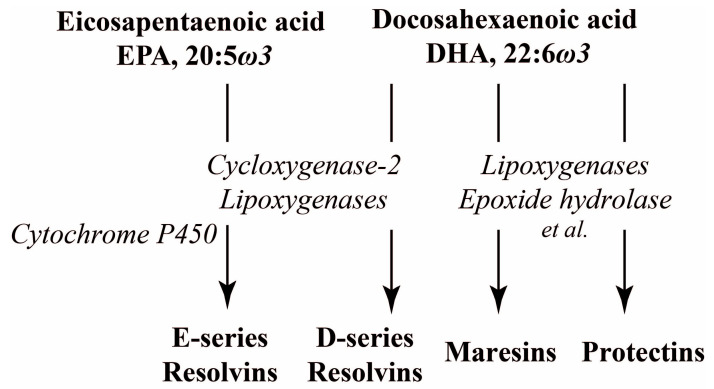
An outline of biosynthetic pathways of the main SPM families.

The diversity of the anti-inflammatory effects of DHA/EPA in the CNS may also be explained by the involvement of their various oxygenated metabolites. PUFAs can undergo oxidation through the action of cytochrome P450 monooxygenase, cyclooxygenase, lipoxygenase, and epoxide hydrolase, operating by one or in combination, in multistep biochemical reactions [17,179,180]. During the resolution phase of inflammation, the substrate preference of these enzymes shifts from ω-6 ARA, whose oxygenated products are highly pro-inflammatory eicosanoids, to ω-3 PUFAs, such as EPA, DPA, and DHA. This shift results in the production of multiple ω-3 PUFA derivatives, named specialized pro-resolving mediators (SPMs), which constitute the individual families, including resolvins (Rvs), protectins (such as, for example, neuroprotection D1) and maresins (MaRs) (Figure 3).

SPMs manage the resolution of inflammation in an autocrine/paracrine manner by coupling with their specific receptors, mainly G-protein-coupled ones [17,180]. Both astrocytes and brain endothelial cells express a wide range of receptors for SPMs [17,181], indicating that SPMs might be important mediators for the resolution of the inflammation in the BBB. In a number of models of brain injury, administration of SPMs has been shown to promote the resolution of inflammation, improve cognitive functions, suppress astrocyte and microglia activation, and augment the expression of inflammatory factors, NF-κB activation and BBB impairment [114,182,183,184,185,186,187,188]. Under various pathological conditions, the expression of SPM receptors was found to increase sharply [17,181,188,189,190].

In rats with chronic cerebral hypoperfusion, MaR1 injected intrathecally, which allows its high concentration in the CNS to be achieved, improved cognitive impairment [185]. It was found that this effect depends on inflammatory resolution, decreased astrocyte activation, and effective protection of the BBB’s structural integrity. MaR1 increased ZO-1 and claudin-5 expression and decreased MMP9 levels [185]. It was suggested that Mar1 interacts with the retinoic acid receptor-related orphan receptor α (RORα) [17], which positively regulates TJ protein claudin domain-containing 1 mRNA expression, interacting with the promoter region of the *CLDND1* gene [191].

In a mouse model of traumatic brain injury, resolvin D1 ameliorated cognitive impairment, suppressed gliosis and restored BBB integrity in the hippocampus by preventing mitochondria dysfunction in astrocytes [188]. RvD1 preserved mitochondrial morphology and membrane potential, reduced mitoROS accumulation and increased mitophagy by activating its target receptor, ALX4/FPR2, as was shown in primary cultured astrocytes [188]. Interestingly, ALX/FPR2 was strongly induced exclusively in reactive astrocytes after brain injury, but not in the neurons or microglia [188]. In a mouse model of postoperative delirium, it was shown that a single intraperitoneal dose of neuroprotectin D1 abolished surgery-induced astrocyte activation and suppressed BBB leakage by increasing the expression of ZO-1, claudin-5, and occludin [187].

microRNAs are known as post-transcriptional or translational repressors of several genes that regulate inflammation and immunity [192]. The involvement of miR-146b and miR-219a-1-3p has been demonstrated in the resolvin D1-induced improvement of functional recovery in a rat model of remote CNS changes following hemicerebellectomy [190]. Intraperitoneally administered RvD1 inhibited the activation of GFAP+ astrocytes, as well as Iba-1+ microglia, and impaired inflammation-induced neuronal cell death in remote regions. RvD1 was found to significantly decrease the expression of inflammatory genes, such as *TLR4* and the *IL-6 receptor*, by binding to ALX/FPR2 and promoting the upregulation of miR-146b and miR-219a-1-3p [190].

A novel class of neuroprotective lipid mediators termed elovanoids was detected [193,194]. They are derived from very-long-chain ω-3 PUFAs (32:6 and 34:6) that are made by elongation of DHA by the elongase ELOVL4 [195]. In mixed neuronal cultures, it was found that their synthesis was stimulated by oxygen/glucose deprivation or NMDA receptor-mediated excitotoxicity. In a mouse model of ischemic stroke, intraventricular administration of these derivatives reduced infarct volumes, promoted cell survival, modulated astrocyte activity and diminished BBB disruption [195].

A higher production of SPMs may explain the anticipated recovery from inflammation that was observed in *fat-1* transgenic mice, which express the *C. elegans fat-1* gene and are, therefore, capable of converting ω-6 PUFAs to ω-3 [196]. In fact, the elevation of ω-3 PUFAs levels and the ω-3/ω-6 ratio in the brain of *fat-1* mice was small [197], but these mice exhibited a downregulated brain inflammatory response and subsequent cognitive improvement [115,198,199], suggesting a role for increased SPM production.

## 6. Role of ω-3 PUFAs in Amyloid-Beta Clearance Mediated by Astrocyte–Endothelial Cell Couple

A close link between Aβ pathogenesis, astrocyte activation and BBB breakdown was established [39,200,201,202]. In this context, the improvement of the BBB is considered a potential therapeutic strategy for addressing Alzheimer’s disease (AD) and cognitive dysfunction [203]. AD is well-known to be associated with microvascular changes and impaired clearance systems, leading to the extracellular accumulation of Aβ and tau proteins. Astrocytes play an important role in the clearance of Aβ through various mechanisms, such as its enzymatic degradation, glymphatic system functioning and regulation of the efflux of Aβ in cooperation with the brain endothelial cells [204,205].

The transport of Aβ across the BBB is mediated by two transporters: the receptor for advanced glycation end products (RAGE), involved in Aβ influx, and the low-density lipoprotein receptor-related protein 1 (LRP1), which provides the clearance of Aβ from the brain. An imbalance between the influx and efflux of Aβ through RAGE and LRP1 in pathological conditions leads to Aβ accumulation within the brain [206]. Aβ accumulation, in turn, causes BBB leakage associated with upregulation of RAGE and MMPs and downregulation of TJ proteins [202,207]. LRP1 is expressed both in endothelial cells and astrocytes [205,208]. Astrocyte dysfunction enhances RAGE activity and lowers LRP1 activity, promoting BBB impairment due to an imbalance between the influx and efflux of Aβ [209]. Silencing of LRP1 in the endothelium leads to activation of the MMP9 pathway, causing the loss of TJ proteins and the increase in BBB permeability [210]. LRP1 in astrocytes contributes to Aβ degradation by secretion of MMPs and other enzymes [205,211,212], facilitating Aβ clearance in the brain, as it has been shown in primary LRP1-null astrocytes, as well as in astrocytic-LRP1-knockout mice [205].

Astrocytes also play a critical role in the elimination of Aβ and tau proteins through the glymphatic system [204]. This mechanism depends on the presence of water transport channels AQP4 in astrocyte endfeet processes. Knocking out astroglial AQP4 significantly hinders the clearance of soluble Aβ and phosphorylated tau from the rat brain parenchyma [213,214]. Reactive astrogliosis results in the loss of AQP4 polarization to the endfoot process and its relocation into the astrocyte soma [215]. This impairs perivascular fluid flow, causing glymphatic dysfunction and leading to the dysregulation of Aβ clearance by the glymphatic system and via transport across the BBB. This results in the accumulation of Aβ not only in the brain parenchyma, but also in the blood vessel walls. The resulting Aβ accretion can further disrupt the reactivity of perivascular AQP4 channels and aggravate endothelial degeneration, thus closing the vicious feed-forward circle [216]. Meanwhile, BBB disruption and an increase in its permeability trigger astrocyte activation and impair AQP4 polarization, thus disturbing the functioning of the glymphatic system [215,217].

ω-3 PUFAs were found to significantly accelerate Aβ clearance from the brain to the circulation and correspondingly increase plasma Aβ levels, decreasing the number of senile plaques in the hippocampus and cortex of AD transgenic mice [39]. This effect was shown to be based on ω-3 PUFA-induced inhibition of astrocytes and microglia activation and on restoration of the levels of LRP1 expression in capillary endothelial cells, which declined during disease progression [39]. High-dose fish oil supplementation has also been shown to significantly reduce Aβ accumulation in retinal blood vessels in the 5xFAD AD mouse model [109].

The protective effect of DHA against the toxic effects of the Aβ42 peptide was demonstrated in primary cultured astrocytes, endothelial cells, and pericytes isolated from the rat brain. This effect of DHA was accompanied by the attenuation of Aβ42-induced morphological changes and decreased ROS generation in all cell types. Additionally, DHA restored the barrier integrity and function of brain endothelial cells impaired under Aβ42 [218]. However, the authors did not link the observed effect to the activation of astrocytes when explaining the obtained data.

The beneficial effect of ω-3 PUFAs on Aβ clearance via mediating the glymphatic system function and AQP4 polarization has been demonstrated [3,20,24]. Both oral fish oil supplementation and the use of *fat-1* transgenic mice, which endogenously produce elevated levels of ω-3 PUFAs, significantly enhanced the interstitial clearance of Aβ from the brain, providing protection against Aβ-induced injury caused by the intracerebroventricular injection of soluble Aβ [20]. It was clearly demonstrated that both endogenously produced and exogenously delivered ω-3 PUFAs significantly inhibited astrocyte activation. It was accompanied by the promotion of AQP4 polarization towards astrocytic endfeet within the impacted brain regions following Aβ injection. The involvement of AQP4 in these effects of ω-3 PUFAs was further confirmed, as the enhancement of extracellular solute clearance induced by ω-3 PUFAs was lost in Aqp4-knockout mice [20]. The AQP4-dependent beneficial effect of ω-3 PUFAs on the glymphatic system was also shown in mice pretreated with fish oil before introduction of TBI [24]. Thereby, ω-3 PUFAs via multifaceted mechanisms improve glymphatic clearance, reduce Aß42 accumulation, and partially prevent loss of AQP4 polarity.

## 7. Concluding Remarks

The data outlined in this review demonstrate that ω-3 PUFA-induced suppression of astrocyte activation or even promotion of their transition from a pro-inflammatory to a neuroprotective phenotype is a prominent pathway for their protective effect on the BBB’s integrity and, therefore, on brain health as a whole. An extensive and complex signaling network involving various transcription factors and receptors mediates the effects of ω-3 PUFAs and their oxidative derivatives on inflammation and oxidative stress in astrocytes. However, in the case of ω-3 PUFAs, it is particularly difficult to find the precise molecular mechanism underlying their effects or to interpret the data obtained. In our opinion, there is hardly any process in the brain that is unaffected by the action of PUFAs, from either the ω-3 or ω-6 families. ω-3 PUFAs are probably important not only, but apparently not as much, as signaling molecules, but also, being constituent components of the cellular membrane, they participate in the regulation of numerous membrane-associated processes. An increase in the content of highly unsaturated long-chain fatty acids, such as DHA, within the lipid bilayer can lead to the alteration of the basic membrane properties, lipid raft organization and the distribution of membrane-bound proteins between raft/non-raft domains, significantly affecting clustering of signal transduction complexes and even redirecting the signal. The same consequences may arise from DHA deficiency. A clear example of this pattern is the critical importance of the membrane enrichment in DHA for the maintenance of the low level of caveolin-dependent transcytosis in brain endothelial cells [18]. Moreover, a plethora of locally produced PUFA derivatives and their specific signaling, and a diverse set of receptors, either GPCR or nuclear, is another level of complexity to data interpretation, as both the precursors and the products can be active participants.

From the intricate network of the complex intercellular communication between various brain cells, we highlighted only one link, namely, vascular–astrocytes coupling. This choice was largely driven by the exclusively tight and multifaceted interrelationship between astrocytes and brain endothelial cells in both normal and pathological conditions. However, it is obvious that the protective effect of ω-3 PUFAs on the maintenance of BBB integrity could be attributed to their direct antioxidative and anti-inflammatory effects on microvascular endothelial cells from the capillary lumen, as was shown for microbiota-derived short-chain fatty acids [219], or on any other element of the neurovascular unit, which responds to inflammatory stimuli. Data on brain endothelial cells cultured alone evidence that ω-3 PUFAs exert an antioxidant effect and upregulate TJ protein expression [220,221]. However, in inflammatory conditions, the integrity and function of the BBB become predominantly dependent on the reactive astrocytes, which can severely disrupt barrier function, accelerating the pathological process. In such cases, common in many neuroinflammatory diseases, the prevention of astrogliosis and alterations of astroglial polarization imbalance by ω-3 PUFAs becomes crucial for preserving BBB integrity and mitigating brain damage. We suggest that a deeper understanding of the role of ω-3 PUFAs in vascular–astrocyte interactions may provide new insights for advancing astrocyte-targeted therapies for neuroinflammatory diseases.

## Data Availability

No new data were created or analyzed in this study. Data sharing is not applicable to this article.

## Abbreviations

The following abbreviations were used in this manuscript:

α1ACT	α 1-antichymotrypsin
ω-3 PUFAs	ω-3 polyunsaturated fatty acids
Aβ	Amyloid β
AJs	Adherent junctions
ALA	α-linolenic acid
AP-1	Activator protein 1, a transcription factor
AQP4	Aquaporin-4
ARA	Arachidonic acid
ARE	Antioxidant responsive element
CD36	Fatty acid translocase
C1q, C3	Complement component 1q, complement component 3
COX-2	Cyclooxygenase-2
DHA	Docosahexaenoic acid
DPA	Docosapentaenoic acid
EPA	Eicosapentaenoic acid
FABPs	Fatty acid-binding proteins
FATPs	Fatty acid transport proteins
GDNF	Glial-derived neurotrophic factor
GFAP	Glial fibrillary acidic protein
GPCR	Gap junction
GPCR	G-protein-coupled receptor
GPR120, GPR 40	G-protein coupled receptors 120 and 40
HO-1	Heme oxygenase 1
ICAM-1	Intercellular adhesion molecule 1
IFN-γ	Interferon-γ
iP	Immunoproteasome
iNOS	Inducible NO synthase
JAM-A	Junctional adhesion molecule
JAK2	Janus kinase 2
IL-1β, IL-6 etc.	Interleukin 1 beta, interleukin 6
LCN2	Lipocalin-2
LPS	Lipopolysaccharide
LRP1	Low-density lipoprotein receptor-related protein 1
LysoPC	Lysophosphatidylcholine
Mfsd2	Major facilitator superfamily domain-containing protein 2
MMPs	Matrix metalloproteinases
NF-κB	Nuclear factor-κb
NQO-1	NAD(P)H dehydrogenase (quinone) 1
Nrf2	Nuclear factor erythroid 2-related factor 2
NRLP3	NLR family pyrin domain-containing 3
PPARs	Peroxisome proliferator-activated receptors
RAGE	Receptor for advanced glycation end-products
RXR	Retinoid X receptor
ROS	Reactive oxygen species
Shh	Sonic hedgehog protein
SOD	Superoxide dismutase
SPMs	Specialized pro-resolving mediators
STAT3	Signal transducer and activator of transcription 3
TGF-β	Transforming growth factor beta
TJs	Tight junctions
TNF-α, TGF-β	Tumor necrosis factor-α, tumor necrosis factor-β
VCAM-1	Vascular cell adhesion molecule 1
ZO-1	Zonula occludens 1 protein
